# A novel heart rate variability based risk prediction model for septic patients presenting to the emergency department

**DOI:** 10.1097/MD.0000000000010866

**Published:** 2018-06-18

**Authors:** Mas’uud Ibnu Samsudin, Nan Liu, Sumanth Madhusudan Prabhakar, Shu-Ling Chong, Weng Kit Lye, Zhi Xiong Koh, Dagang Guo, R. Rajesh, Andrew Fu Wah Ho, Marcus Eng Hock Ong

**Affiliations:** a Duke-NUS Medical School, National University of Singapore; b Health Services Research Centre, Singapore Health Services; c Yong Loo Lin School of Medicine, National University of Singapore; d Department of Emergency Medicine, KK Women's and Children's Hospital; e Department of Emergency Medicine, Singapore General Hospital; f SingHealth Emergency Medicine Residency Program, Singapore.

**Keywords:** emergency department, heart rate variability, risk prediction, sepsis

## Abstract

A quick, objective, non-invasive means of identifying high-risk septic patients in the emergency department (ED) can improve hospital outcomes through early, appropriate management. Heart rate variability (HRV) analysis has been correlated with mortality in critically ill patients. We aimed to develop a Singapore ED sepsis (SEDS) predictive model to assess the risk of 30-day in-hospital mortality in septic patients presenting to the ED. We used demographics, vital signs, and HRV parameters in model building and compared it with the modified early warning score (MEWS), national early warning score (NEWS), and quick sequential organ failure assessment (qSOFA) score.

Adult patients clinically suspected to have sepsis in the ED and who met the systemic inflammatory response syndrome (SIRS) criteria were included. Routine triage electrocardiogram segments were used to obtain HRV variables. The primary endpoint was 30-day in-hospital mortality. Multivariate logistic regression was used to derive the SEDS model. MEWS, NEWS, and qSOFA (initial and worst measurements) scores were computed. Receiver operating characteristic (ROC) analysis was used to evaluate their predictive performances.

Of the 214 patients included in this study, 40 (18.7%) met the primary endpoint. The SEDS model comprises of 5 components (age, respiratory rate, systolic blood pressure, mean RR interval, and detrended fluctuation analysis α2) and performed with an area under the ROC curve (AUC) of 0.78 (95% confidence interval [CI]: 0.72–0.86), compared with 0.65 (95% CI: 0.56–0.74), 0.70 (95% CI: 0.61–0.79), 0.70 (95% CI: 0.62–0.79), 0.56 (95% CI: 0.46–0.66) by qSOFA (initial), qSOFA (worst), NEWS, and MEWS, respectively.

HRV analysis is a useful component in mortality risk prediction for septic patients presenting to the ED.

## 1 Introduction

Sepsis is highly prevalent and increasing in incidence,^[1]^ accounting for a large proportion of admissions to the intensive care unit (ICU) with high hospital costs and a 10% to 20% in-hospital mortality (IHM) rate.^[2–8]^ Early identification and therapy of septic patients have been shown to reduce healthcare expenditures, hospital length of stay, and mortality.^[9–15]^ Most septic cases present in the emergency department (ED) and on the wards rather than the ICU.^[16]^ Given the limited resources in the ED setting, it would be prudent to quickly identify patients who have a higher mortality risk from sepsis, so that more attention can be given and timely interventions for preventable and treatable complications can be made.^[17]^

A number of clinical tools have been developed to risk stratify septic patients which typically combine clinical variables to estimate the risk of short-term mortality.^[18]^ These include physiological scoring systems such as the acute physiology and chronic health evaluation (APACHE),^[19]^ simplified acute physiology score (SAPS),^[20]^ sequential organ failure assessment score (SOFA),^[21]^ and multiple organ dysfunction score (MODS).^[22]^ However, these tools were not created for the ED setting, and the information required for scoring, such as laboratory investigations, is not readily available in the ED. The rapid emergency medicine score (REMS)^[23]^ and the mortality in emergency department sepsis (MEDS)^[24]^ score were developed to predict the IHM for septic ED patients but have shown mixed results which warrants further clinical evaluation of their performance.^[25–27]^ The recently derived and validated quick SOFA (qSOFA) score was externally validated among septic patients presenting to the ED using the worst level of the 3 components of qSOFA during ED stay to compute the score and showed good prognostic accuracy for IHM.^[28]^ However, another recent study by Churpek et al^[29]^ showed that commonly used early warning scores such as the national early warning score (NEWS) and the modified early warning score (MEWS)^[30]^ were more accurate than qSOFA in predicting mortality in patients with suspected infection presenting to the ED.^[29]^ Hence, it remains to be seen if there is a more reliable tool that utilizes novel, objective, and quickly attainable predictors to help clinicians in the ED to risk stratify septic patients early and with a more consistent predictive accuracy.

Heart rate variability (HRV) analysis provides a quick, objective, and non-invasive method of evaluating autonomic modulation of the cardiovascular system.^[31]^ It is a technique that examines the beat-to-beat variation in heart rate. Septic patients have reduced sympathovagal balance and impaired sympathetic activity, which lead to varying degrees of cardiac autonomic dysfunction.^[32]^ This phenomenon can be detected by HRV analysis as HRV measures were shown to be independent predictors of early deterioration, and increased morbidity and mortality, including in the ED setting.^[33–38]^ HRV analyses are divided into linear and non-linear methods.^[31]^ Linear methods include HRV parameters measured in time-domain or frequency-domain. Time-domain HRV parameters are statistical calculations of consecutive RR time intervals (known as NN intervals in HRV terms), and how they are correlated with each other. Frequency-domain HRV parameters are based on spectral analysis. Recent studies suggested that the regulation of the cardiovascular system interact with each other in a nonlinear way^[39–41]^ and the HRV analysis using non-linear methods reflect these mechanisms.^[42]^ Many studies have highlighted the potential role of HRV as an independent predictor of mortality and an important component of a risk prediction model in patients presenting with chest pain^[43–47]^ and trauma.^[48–52]^ Several studies have also shown that certain individual HRV parameters can provide an early indication of illness severity either as a progression to septic shock or IHM, in septic patients presenting to the ED^[33–35]^ and in the ICU.^[53]^ However, to date, no study has developed a risk assessment model for sepsis in the ED that incorporates HRV measures together with patient demographics and traditional vital signs. Therefore, HRV could potentially be incorporated into an early risk assessment tool to reliably predict mortality in septic patients presenting to the ED.

In this study we aim to develop a novel risk assessment model (henceforth referred to as the Singapore Emergency Department Sepsis [SEDS] model) that incorporates quickly attainable parameters including patient demographics, vital signs, and HRV measures to predict mortality and other serious adverse events such as intubation and ICU admission in septic patients presenting to the ED. We hypothesize that the SEDS model will outperform the currently available scoring systems namely the NEWS, MEWS, and qSOFA score, at predicting 30-day IHM and a composite outcome of adverse events including IHM, intubation, and ICU admission, within 30 days of a septic patient presenting to the ED.

## 2 Methods

### 2.1 Design and setting

We conducted a retrospective analysis on data collected from a convenience sample of patients between September 2014 and April 2016. The study was performed at the ED of the Singapore General Hospital (SGH), a tertiary care hospital in Singapore. The SGH ED sees between 300 and 500 patients a day. All patients were triaged on arrival by a trained nurse and subsequently seen by an emergency physician. Triage is performed by nurses using the national Singaporean patient acuity category scale (PACS), a symptom-based triage system without strict physiological criteria. ED patients are classified with a PACS score, which ranges from 1 to 4 and represents the degree of urgency in patient attendance. Patients with PACS 1 are the most critically ill, those with PACS 2 are non-ambulant, those with PACS 3 are ambulant, and those with PACS 4 are non-emergencies. Our study focused on patients presenting with sepsis, who were triaged to either PACS 1 or 2 units where they received further ECG monitoring. The study was approved by SingHealth Centralised Institutional Review Board (Ref: 2016/2858) with a waiver of patient consent.

### 2.2 Patient recruitment and eligibility

All patients clinically suspected to have sepsis and met at least 2 of the 4 Systemic Inflammatory Response Syndrome (SIRS) criteria^[54]^ were included in this study. The SIRS criteria are temperature (<36 °C or >38 °C), heart rate (>90 beats/min), respiratory rate (>20 breaths/min), and total white count (<4000/mm^3^ or >12,000/mm^3^). The vital signs and HRV parameters used in this study were recorded when the patient was triaged.

### 2.3 Data collection

Patient demographics and first vital signs recorded in the patients’ electronic medical record collected either in triage or the ED were used for analysis. Five to 6 minutes one-lead electrocardiogram (ECG) tracings were obtained from X-Series Monitor (ZOLL Medical Corporation, Chelmsford, MA). Subsequently, ECG tracings were loaded into the Kubios HRV program version 2.2 (Kuopio, Finland).^[55]^ The program automatically detected QRS complexes, but each ECG was also manually screened to ensure QRS detection was correct. The position of the QRS detector was adjusted if misplaced. The R-R interval time series was then screened for rhythm, artifacts, and ectopic beats. If artifacts or ectopic beats were few (<5), they were removed from the R-R interval time series with remaining segments spliced together for analysis. HRV measures of time domain and frequency domain along with non-linear variables were computed.

### 2.4 Outcomes

The primary outcome was IHM within 30 days of ED admission. Secondary outcome was a composite outcome including IHM, intubation or admission to the ICU, within 30 days of ED admission.

### 2.5 Statistical analyses

Statistical analysis was performed using SPSS version 23 (IBM corporation, Armonk, NY). Continuous variables were presented as means (standard deviation) while categorical variables were presented as numbers (percentage). Patient demographics, comorbidities, drug history, vital signs, and HRV measures were compared between the group of patients who met the primary outcome and the group of patients who did not using the independent two-tailed *t* test for continuous variables and the chi-square test or the Fisher exact test, as appropriate, for categorical variables, with a statistically significant difference defined as *P* < .05.

### 2.6 Predictive model

To build the predictive Singapore Emergency Department Sepsis (SEDS) model, objective variables obtainable within 6 minutes and without chart review were considered as possible covariates. A total of 22 HRV parameters, 6 vital signs, and 3 demographic variables (age, sex, and ethnicity) were screened for candidate predictors of 30-day IHM.

First, a univariate analysis was done comparing each of these possible covariates between patients who did and did not meet the primary outcome. Variables with *P* < .20 or deemed to be clinically relevant were entered as covariates and the primary 30-day IHM outcome as the dependent variable, in a forward selection stepwise logistic regression model. The retained covariates were used to construct the SEDS model. The predictive performance of the SEDS model was assessed using the receiver operating characteristic (ROC) analysis.

### 2.7 qSOFA, news, and mews scores

For a complete comparison, the qSOFA, NEWS, and MEWS scores were also computed. Methods for calculating the qSOFA score, NEWS score, and MEWS score have been defined in prior original articles.^[30,56,57]^ The variables for each score were obtained using the first available recorded reading for vital signs. An additional qSOFA score was also calculated for each patient based on the worst level for each component of the score during the ED stay as described by Freund et al.^[28]^

### 2.8 Model comparisons

The predictive performance of the SEDS model was compared with the qSOFA (both initial and worst parameters in ED), MEWS, and NEWS score based on the area under the ROC curve (AUC) for the primary 30-day IHM outcome and the 30-day composite outcomes.

## 3 Results

### 3.1 Patient enrollment

**Figure 1:**
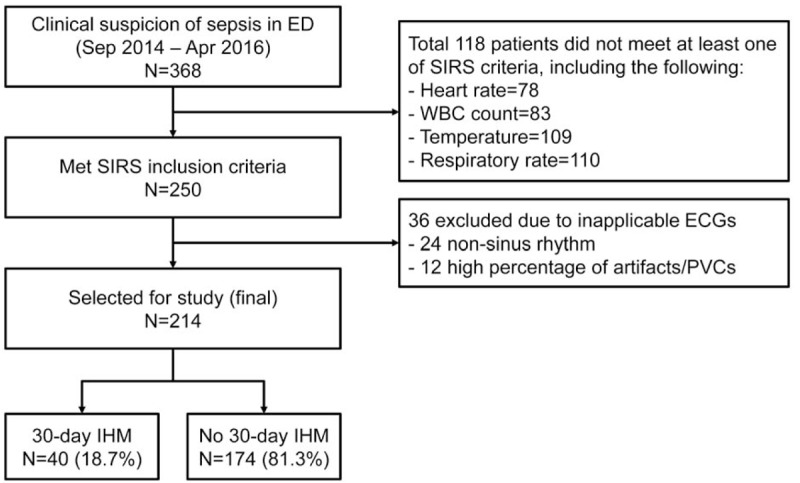
Patient flow with breakdown of 30-day in-hospital mortality (IHM) outcome.

A total of 368 patients with a clinical suspicion were enrolled and 118 were excluded for not meeting the Systemic Inflammatory Response Syndrome. Thirty-six patients were further removed due to inapplicable ECG readings either having non-sinus rhythm, or high proportion of artifacts or premature ventricular complexes. Two hundred fourteen patients were included for the final analysis. The patient selection flow is shown in Fig. 1.

### 3.2 Outcomes

**Figure 2:**
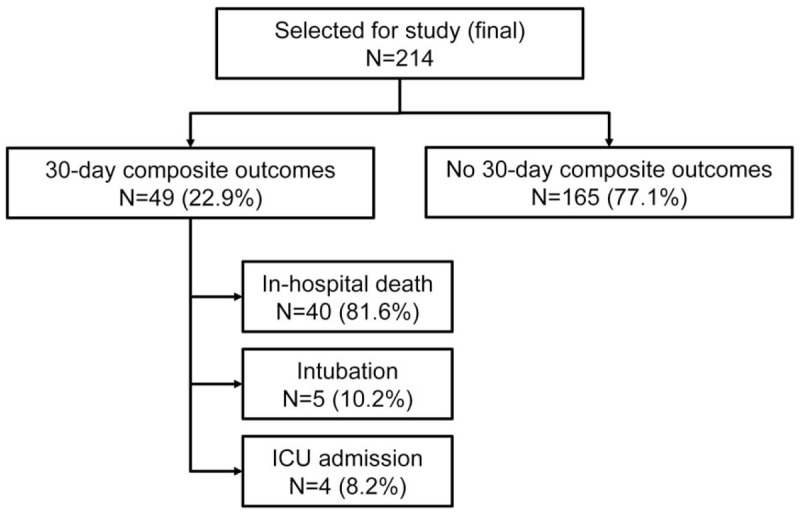
Breakdown of the composite outcomes.

Of the 214 patients, 40 (18.7%) met the primary 30-day IHM outcome and 49 (22.9%) met the 30-day composite outcomes. Figure 1 shows the breakdown of patients who met the primary outcome. Figure 2 shows the breakdown of patients who met the secondary outcome, including 40 in-hospital deaths (81.6%), 5 intubated patients (10.2%), and 4 ICU admissions (8.2%).

### 3.3 Baseline characteristics

Table 1 shows the baseline characteristics of the patients who did and did not meet the primary 30-day IHM outcome. The patients who met the primary outcome (mean age 75 years old) were older than the patients who did not meet the primary outcome (mean age 65 years old). There was no difference in the proportion of men and ethnicity distribution among the 2 groups. While there was no difference in triaging both group of patients into high risk categories based on the PACS assigned to the patient, there was a difference in the patient disposition of the patients from the emergency department with a higher percentage of patients who met the primary outcome requiring subsequent management in the ICU. The presence of several comorbidities was also compared between the 2 groups. A serious infection was defined as any previous hospitalization for any infection or sepsis. The past medical history which includes comorbidities and a serious infection, showed no difference between the 2 groups. The use of medications that are known to affect heart rate variability was also not different between the 2 groups. There was also no overall difference in the source of infection for sepsis in the 2 patient groups.

**Table 1:**
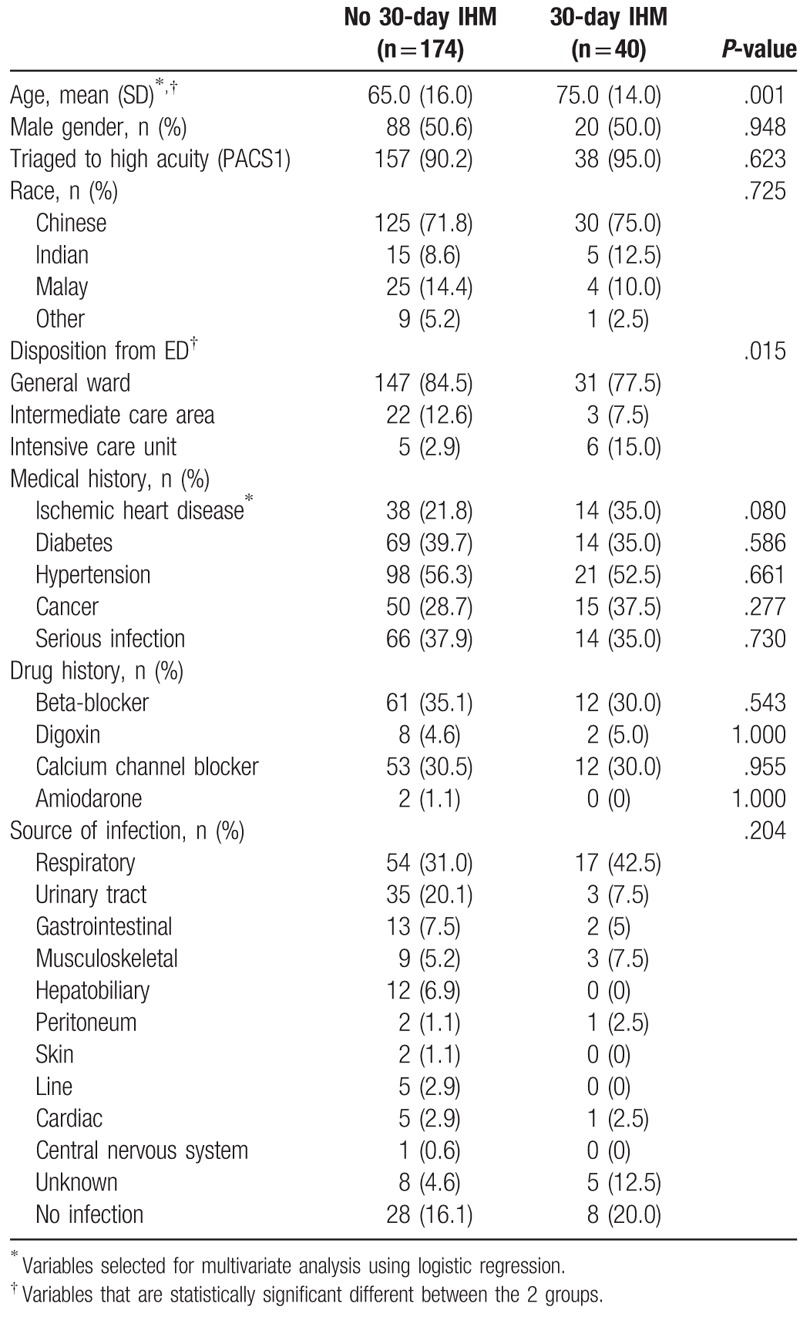
Baseline characteristics and clinical parameters of patients by presence and absence of 30-day in-hospital mortality (IHM).

### 3.4 Univariable statistical analysis

Table 2 shows vital signs and HRV variables compared between patients who did and did not meet the primary outcome. Among vital signs, Glasgow Coma Scale (GCS) was higher in patients who met the primary outcome while respiratory rate was higher in patients who did not meet the primary outcome. For time domain HRV parameters, standard deviation of NN (between consecutive R waves on ECG) time intervals (SD NN), standard deviation of heart rate (SD HR), root mean square of the differences between adjacent NN intervals (RMSSD) and the baseline width of the minimum square difference triangular interpolation of the highest peak of the histogram of all NN intervals (TINN) showed significant differences between the 2 groups. Within the frequency domain, only the normalized low frequency (LF) power and normalized high frequency (HF) power were significantly different between the 2 groups. Of the non-linear HRV variables, Poincare plot SD1, Poincare plot SD2, and detrended frequency analysis (DFA) α2 were significantly different in the 2 groups.

**Table 2:**
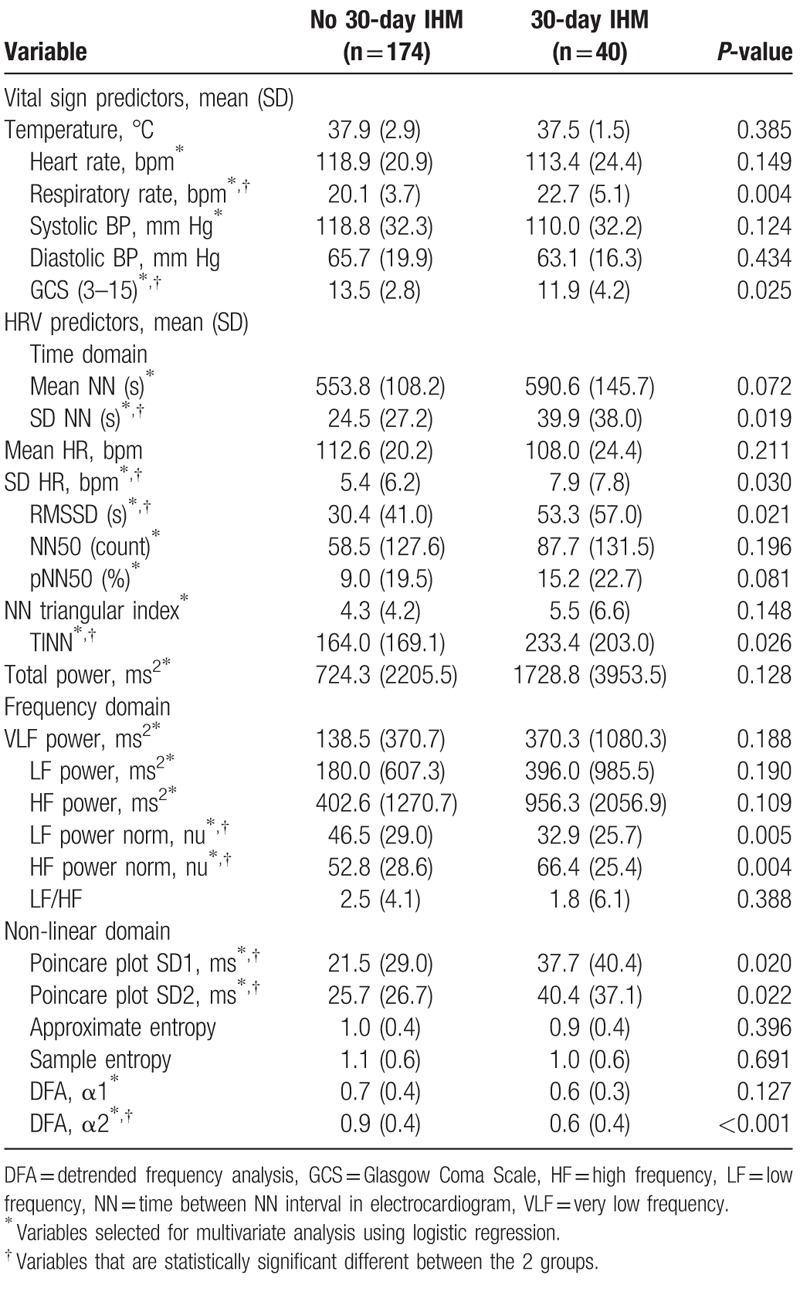
Vital signs and heart rate variability (HRV) variables of patients who did and did not meet the 30-day in-hospital mortality (IHM) outcome.

### 3.5 SEDS model

Table 3 shows the final 5 variables included in the SEDS model with their corresponding adjusted odds ratios, for predicting 30-day IHM. Variables include 1 demographic characteristic (age), 2 vital signs (respiratory rate, systolic blood pressure), and 2 HRV variables (mean NN, DFA α2). Table 4 shows final 4 variables included in the SEDS model with their corresponding adjusted odds ratios, for predicting composite outcome of IHM, intubation or admission to the ICU within 30 days of ED admission. Variables include 3 vital signs (respiratory rate, systolic blood pressure, GCS) and 1 HRV variable (DFA α2).

**Table 3:**
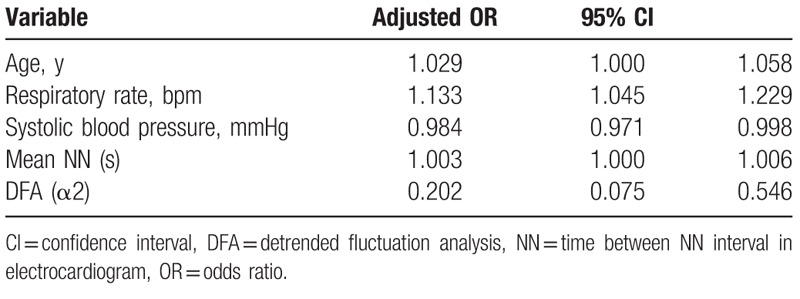
Odds ratios of covariates remaining in SEDS model for predicting 30-day in-hospital mortality (IHM) following forward selection stepwise logistic regression.

**Table 4:**
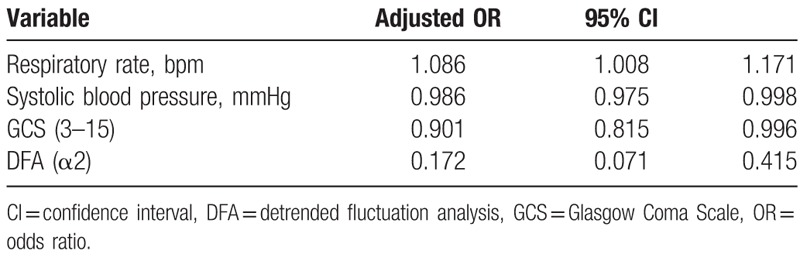
Odds ratios of covariates remaining in SEDS model for predicting composite outcomes following forward selection stepwise logistic regression.

### 3.6 Prediction of 30-day IHM outcome

**Figure 3:**
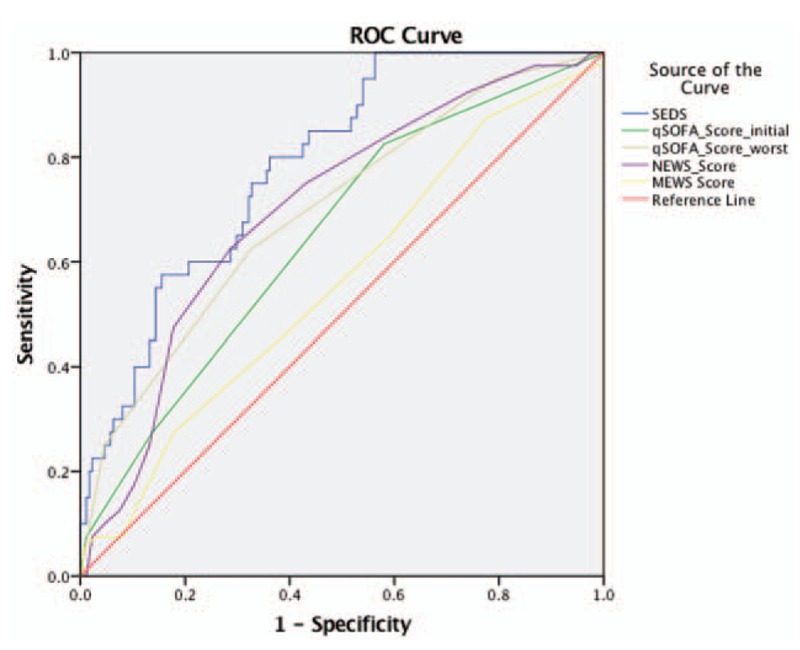
Predictive performances of the SEDS model and the different scoring systems represented by receiver operating characteristic (ROC) curves for prediction of 30-day IHM. IHM = in-hospital mortality; SEDS = Singapore emergency department sepsis.

Figure 3 shows the ROC curves while Table 5 lists the AUC values of the SEDS model, qSOFA score based on initial and worst parameters in the ED as well as for NEWS and MEWS in predicting the primary outcome of 30-day IHM. SEDS model had the highest c-statistic at 0.79 followed by both qSOFA (worst) and NEWS at 0.70. The qSOFA (initial) and MEWS had c-statistics of 0.65 and 0.56, respectively.

**Table 5:**
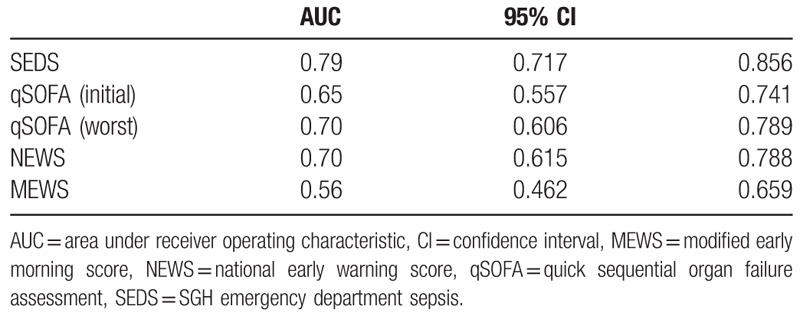
Predictive performances of SEDS model and the different illness scoring systems—qSOFA, NEWS, and MEWS for predicting 30-day in-hospital mortality (IHM).

### 3.7 Prediction of 30-day composite outcomes

**Figure 4:**
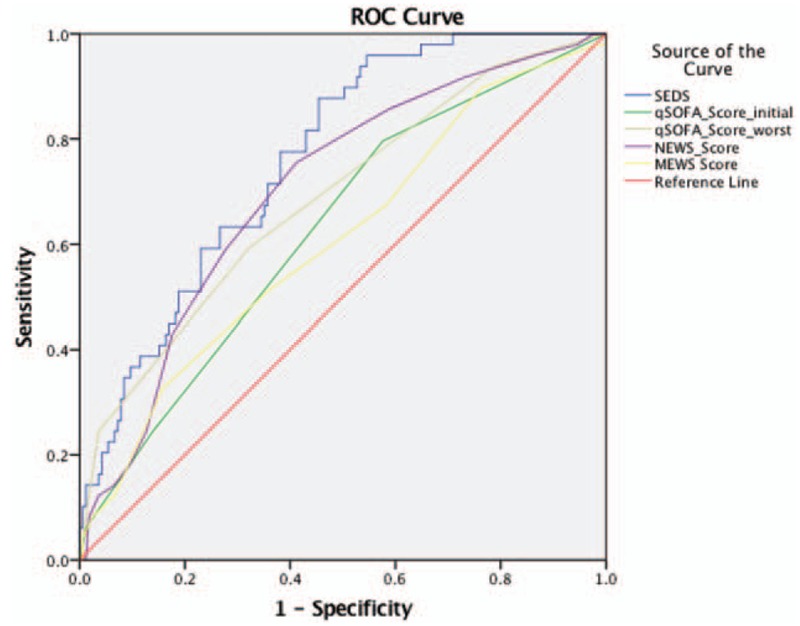
Predictive performances of the SEDS model and the different scoring systems represented by receiver operating characteristic (ROC) curves for prediction of composite outcomes. SEDS = Singapore emergency department sepsis.

Figure 4 shows the ROC curves while Table 6 lists the AUC values of the SEDS model, qSOFA score based on initial and worst parameters in the ED as well as for NEWS and MEWS in predicting the composite outcomes (in-hospital death, intubation, ICU admission) within 30 days of ED admission. SEDS model had the highest c-statistic at 0.76 followed by NEWS and qSOFA (worst) at 0.70 and 0.69, respectively. The qSOFA (initial) and MEWS had c-statistics of 0.63 and 0.61, respectively.

**Table 6:**
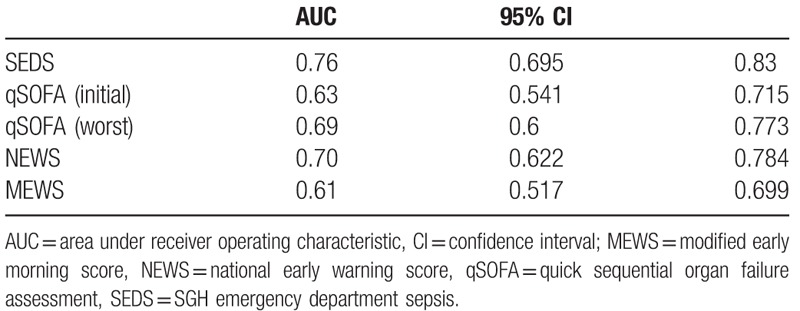
Predictive performances of SEDS model and the different illness scoring systems—qSOFA, NEWS, and MEWS for predicting the composite outcomes.

## 4 Discussion

In this observational cohort study, we constructed a risk assessment model (SEDS model) using demographic data, vital signs, and HRV parameters for the prediction of 30-day IHM in septic patients in the ED. The SEDS model, which only utilizes objective and quickly attainable parameters, includes age, 2 vital signs (respiratory rate and systolic blood pressure), and 2 HRV parameters (mean NN and DFA α2). In terms of AUC, our model significantly outperformed qSOFA (worst), qSOFA (initial), NEWS and MEWS score (0.79 vs 0.70, 0.65, 0.70, and 0.56, respectively) in predicting the primary outcome of 30-day IHM. In addition, for the prediction the composite outcomes of 30-day IHM, intubation and ICU admission, our model also outperformed qSOFA (worst), qSOFA (initial), NEWS and MEWS score (AUC: 0.76 vs 0.69, 0.63, 0.70, and 0.61, respectively).

Vital signs are well-established and frequently employed during clinical risk prediction in the ED.^[23,24,58,59]^ The 3 comparators used in this study were qSOFA, NEWS, and MEWS. All of them utilize traditional vital signs as part of their score. The recently derived and validated qSOFA score requires 3 parameters, all of which are routinely measured vital signs (systolic blood pressure, respiratory rate, and altered mental status) for the prediction of mortality in septic patients in the ED. Our model incorporates 2 vital signs (systolic blood pressure and respiratory rate) which are components of qSOFA, NEWS, and MEWS scores, affirming the strong predictive value of these parameters further. However, altered mental status as measured by the GCS was excluded in our model. This is possibly due to the potential subjectivity of such a measure as studies have shown only a moderate degree of interrater agreement using GCS on patients including those in the ED.^[60,61]^ One study also showed that in addition to GCS, the Alert, Voice, Pain, Unresponsive (AVPU) score which is part of the NEWS and MEWS score also had poor interrater agreement.^[62]^

One possible explanation to the poor predictive performance of qSOFA in this study as compared with other studies is the different inclusion criteria.^[28,29,63,64]^ Our study included patients with a clinical suspicion of sepsis by the ED physician and meeting at least 2 of the SIRS criteria while others included only patients with administered intravenous antibiotics, blood cultures investigation, or confirmed source of infection.^[28,29]^ In 1 study that used a similar inclusion criteria to our study, the authors reported that the predictive performance of qSOFA was comparable to ours in terms of AUC in predicting mortality.^[64]^ Despite being sensitive and nonspecific, the SIRS criteria have been recommended to be a major part of sepsis diagnosis since its development in 1991.^[54,65]^ While the SIRS definition of sepsis has recently been replaced with a new state of sepsis, defined as a life threatening organ dysfunction caused by a dysregulated host response to infection, the usefulness of the SIRS criteria for the identification of infection was still emphasized by the same task force.^[56,66]^ Furthermore, the primary endpoint differences across the studies on qSOFA in septic patients presenting to the ED and our study could also account in part for the predictive performance inconsistency of qSOFA. We used patient mortality within 30 days that occurred in hospital during the same admission when the vitals and HRV parameters of the patients were taken and sepsis suspected at the ED. Other studies either did not specify a time period for mortality^[30]^ or used a similar period for mortality without specifying if it is strictly within the same admission or not.^[64]^ We chose 30-day IHM, similar to Freund et al,^[28]^ as our primary endpoint because this outcome is more likely to be sepsis-related compared with an out-of-hospital mortality or mortality from a subsequent hospital admission and hence, more relevant to the purpose of our study. It is also more meaningful for physicians in the ED as well as the wards in terms of administering possible consequential interventions such as closer monitoring and less conservative management of high risk patients. However, Freund et al^[28]^ differ from our study since they collected the worst levels of the 3 parameters during the patients’ ED stay in computing the qSOFA score (namely highest respiratory rate, lowest systolic blood pressure, and lowest GCS score). This method of calculating the qSOFA score may not be consistent across patients as it directly depends on their length of stay in the ED and is not practical for eventual implementation since risk stratification of septic patients in the ED should be done as early as possible.^[9–15,17]^ Hence, a standardized time of parameter measurements taken at the initial presentation of the patients to the ED would be most practical and was done in our study. Finally, this is the first time these scores are being validated in Singapore and ethnic differences may account for part of the disparity in predictive performances observed in this study and the literature.

To improve the predictive performance of these risk stratification tools, many studies have reported the clinical and prognostic value of HRV parameters in the evaluation of patients with sepsis presenting to the ED.^[33–35,67]^ However, they have yet to be applied clinically. Our study demonstrates that HRV parameters can be successfully implemented in risk stratification of septic patients presenting to the ED, together with other established, traditional prognosticators. Although this is the first study showing the significance of the time-domain HRV parameter mean NN, which is the average of all the intervals between heartbeats or consecutive R waves on ECG,^[31]^ as a potential measure of risk assessment in an illness, several studies have shown the diagnostic and prognostic value of DFA in coronary heart disease.^[68–71]^ DFA is a non-linear method of HRV analysis which quantifies the self-similarity of signals using the fractal property.^[42,72,73]^ In simple terms, DFA measures the long range correlation patterns of the R-R interval time series which includes a short-term and long-term fractal scaling exponent, α1 and α2, respectively. The degree of fractal correlation has been shown to reflect sympathetic and parasympathetic tone.^[74]^ However, large, long-term prospective studies need to be done to establish the physiological range of values for each of the HRV parameters. Although a number of studies have reported the effect of endotoxemia on heart rate and HRV, attributed to autonomic dysregulation,^[75–77]^ the underlying physiological interactions are complex and are not well understood.^[32]^ In neonatal literature, HRV parameters have shown usefulness in identifying the presence of sepsis even before a clinical diagnosis,^[78,79]^ and these findings have translated to clinical tools in sepsis prediction.^[80]^ In general, our findings deserve further investigations thus more research works would be useful in studying the electrophysiology and the interpretations of HRV parameters, which will ultimately help in clinical decision-making by physicians.

This study has several limitations. Firstly, this was a single-center investigation in a tertiary Singapore hospital with a small sample size and low event rate. Therefore, the results may not be generalizable to other settings and larger multicenter prospective studies are required to validate our results. Currently, as a follow-up to this study, we are prospectively recruiting more patients to conduct a larger similar study. Secondly, there is no gold standard to determine when a patient is septic and we had enrolled patients who had clinically diagnosed sepsis based on criteria different from several other similar studies, so we might have excluded patients who were septic and included others who were not. Moreover, because of the inherent nature of HRV in measuring variations in heart beats, patient ECGs with non-sinus rhythm, high proportion of artifacts, or premature ventricular complexes were excluded from this study which could represent a form of data loss. Finally, even though HRV parameters are objective, quickly attainable and predictive of adverse outcomes in septic patients as shown in this study, we acknowledge that HRV parameters cannot be manually calculated or interpreted from a patient's ECG at the bedside, and this may limit the application of HRV technology in centers without the necessary monitoring devices. We are currently developing a portable hardware device to integrate data acquisition and analysis. We believe that such a device will help clinicians quickly identify septic patients at high risk of developing adverse events such as death, ICU admission, and intubation.

## 5 Conclusions

In this study, we derived a novel risk assessment model (i.e., SEDS) which incorporates age, 2 vital signs (respiratory rate and systolic blood pressure), and 2 HRV parameters (mean NN and DFA α2) for septic patients presenting to the ED. Our model outperformed qSOFA, NEWS, and MEWS score in predicting mortality and other adverse events such as intubation and ICU admission. Larger studies will be needed to establish a risk assessment score from this model and validate its predictive performance in other independent samples.

## Acknowledgments

The authors would like to thank and acknowledge all contributions made from doctors, nurses, and research assistants from the Department of Emergency Medicine, Singapore General Hospital.

## Author contributions

**Conceptualization:** Nan Liu, Marcus Eng Hock Ong.

**Data curation:** Mas’uud Samsudin, Nan Liu, Weng Kit Lye.

**Formal analysis:** Mas’uud Samsudin, Nan Liu, Weng Kit Lye.

**Funding acquisition:** Mas’uud Samsudin, Nan Liu, Marcus Eng Hock Ong.

**Investigation:** Mas’uud Samsudin, Nan Liu, Sumanth Prabhakar, Shu-Ling Chong, Weng Kit Lye, Zhi Xiong Koh, Dagang Guo, R Rajesh, Andrew Ho, Marcus Eng Hock Ong.

**Methodology:** Mas’uud Samsudin, Nan Liu, Sumanth Prabhakar, Shu-Ling Chong, Weng Kit Lye, Zhi Xiong Koh, Dagang Guo, R Rajesh, Andrew Ho, Marcus Eng Hock Ong.

**Project administration:** Mas’uud Samsudin, Nan Liu, Sumanth Prabhakar, Shu-Ling Chong, Weng Kit Lye, Zhi Xiong Koh, Dagang Guo, R Rajesh, Andrew Ho, Marcus Eng Hock Ong.

**Resources:** Mas’uud Samsudin, Nan Liu, Sumanth Prabhakar, Shu-Ling Chong, Weng Kit Lye, Zhi Xiong Koh, Dagang Guo, R Rajesh, Andrew Ho, Marcus Eng Hock Ong.

**Software:** Mas’uud Samsudin, Nan Liu, Shu-Ling Chong, Weng Kit Lye, Zhi Xiong Koh, Dagang Guo, R Rajesh, Andrew Ho.

**Supervision:** Nan Liu, Marcus Eng Hock Ong.

**Validation:** Mas’uud Samsudin, Nan Liu, Sumanth Prabhakar, Shu-Ling Chong, Weng Kit Lye, Zhi Xiong Koh, Dagang Guo, R Rajesh, Andrew Ho, Marcus Eng Hock Ong.

**Visualization:** Mas’uud Samsudin, Nan Liu, Sumanth Prabhakar, Shu-Ling Chong, Weng Kit Lye, Zhi Xiong Koh, Dagang Guo, R Rajesh, Andrew Ho, Marcus Eng Hock Ong.

**Writing – review and editing:** Mas’uud Samsudin, Nan Liu, Sumanth Prabhakar, Shu-Ling Chong, Weng Kit Lye, Zhi Xiong Koh, Dagang Guo, R Rajesh, Andrew Ho, Marcus Eng Hock Ong.

**Writing – original draft:** Mas’uud Samsudin, Nan Liu, Sumanth Prabhakar, Shu-Ling Chong, Weng Kit Lye, Zhi Xiong Koh, Dagang Guo, R Rajesh, Andrew Ho, Marcus Eng Hock Ong.
